# 39. VIRUSES AND SCHIZOPHRENIA: IMPLICATIONS FOR PATHOPHYSIOLOGY AND TREATMENT

**DOI:** 10.1093/schbul/sby014.158

**Published:** 2018-04-01

**Authors:** Alan Breier

**Affiliations:** Indiana University School of Medicine

## Abstract

**Overall Abstract:** The viral hypothesis of schizophrenia posits that viral infections disrupts cortical circuits that give rise to schizophrenia psychopathology. Prenatal viral exposure during key neurodevelopmental periods, either through direct effects on fetal brain or exposure to excessive maternal cytokines and other chemokines, have been implicated. In addition, abnormal activation of dormant neuro-viruses have been linked to the pathophysiology of schizophrenia. Activation of dormant viruses has potentially important treatment implication for therapies, such as valacyclovir, that suppress viral activity. Among the viruses that have been mostly frequently associated with schizophrenia include herpes simplex virus type 1 (HSV1) and Epstein-Barr virus (EBV). The purpose of this symposium is to focus on the role of viruses in the pathophysiology of schizophrenia and results of antiviral treatment trials in this illness.

Diana Perkins will present data from the North American Prodrome Longitudinal Study (NAPLS2) which is an eight-site observational study of predictors and mechanism of conversion to psychosis and is comprised of a cohort of 763 individuals at clinical high risk for developing psychosis. This paper examines methylation of promoter regions of genes associated with gene expression and reports that 10 markers correctly classified individuals who converted to psychosis. The SIRT1 gene, that is upregulated with HSV, was among the predictive markers.

Faith Dickerson will focus on the association between HSV1 exposure and cognitive impairment in schizophrenia and the potential link between EBV and this illness. In a large cohort of 828 individuals and 573 controls, a significant relationship between HSV1 exposure and cognitive impairment was found. The strongest linkage was in the domain of immediate memory. In 397 subjects with schizophrenia who were compared to 289 controls, significantly higher levels of antibodies to the EBV viral capsid antigens (VCA) was discovered in the schizophrenia cohort.

Vishwajit Nimgaonkar will examine the relationship between HSV1 infection and cognitive performance in a mixed cohort of 226 individuals and present the results of a randomized clinical trial of the anti-viral agent valacyclovir. HSV1 infected participants had significantly lower scores on Emotion Identification and Discrimination (EMOD), spatial memory and spatial ability irrespective of schizophrenia diagnoses. Valacyclovir treatment (1.5 grams BID, 16-week trial) improved EMOD.

Alan Breier will report the results of the VISTA study – 12-site, double-blind, placebo controlled, 16-week trial of the anti-viral medication valacyclovir (3 grams/day) in early phase schizophrenia. 170 subjects were randomized of whom 74 were HSV-1 seropositive and 96 were seronegative. Baseline working memory scores (letter number sequence) were significantly lower in HSV1 positive as compared to HSV1 negative subjects. Analysis of valacyclovir treatment outcomes have only recently commenced and are ongoing. The complete data set (cognitive domains, role function, symptoms and safety) will be presented in full at the meeting.

